# AS1411-Induced Growth Inhibition of Glioma Cells by Up-Regulation of p53 and Down-Regulation of Bcl-2 and Akt1 via Nucleolin

**DOI:** 10.1371/journal.pone.0167094

**Published:** 2016-12-01

**Authors:** Ye Cheng, Gang Zhao, Siwen Zhang, Fares Nigim, Guangtong Zhou, Zhiyun Yu, Yang Song, Yong Chen, Yunqian Li

**Affiliations:** 1 Department of Neurosurgery, The First Hospital of Jilin University, Changchun, China; 2 Department of Endocrine, The First Hospital of Jilin University, Changchun, China; 3 Department of Neurosurgery, Harvard Medical School, Boston, United States of America; Swedish Neuroscience Institute, UNITED STATES

## Abstract

AS1411 binds nucleolin (NCL) and is the first oligodeoxynucleotide aptamer to reach phase I and II clinical trials for the treatment of several cancers. However, the mechanisms by which AS1411 targets and kills glioma cells and tissues remain unclear. Here we report that AS1411 induces cell apoptosis and cycle arrest, and inhibits cell viability by up-regulation of p53 and down-regulation of Bcl-2 and Akt1 in human glioma cells. NCL was overexpressed in both nucleus and cytoplasm in human glioma U87, U251 and SHG44 cells compared to normal human astrocytes (NHA). AS1411 bound NCL and inhibited the proliferation of glioma cells but not NHA, which was accompanied with up-regulation of p53 and down-regulation of Bcl-2 and Akt1. Moreover, AS1411 treatment resulted in the G2/M cell cycle arrest in glioma cells, which was however abolished by overexpression of NCL. Further, AS1411 induced cell apoptosis, which was prevented by silencing of p53 and overexpression of Bcl-2. In addition, AS1411 inhibited the migration and invasion of glioma cells in an Akt1-dependent manner. Importantly, AS1411 inhibited the growth of glioma xenograft and prolonged the survival time of glioma tumor-bearing mice. These results revealed a promising treatment of glioma by oligodeoxynucleotide aptamer.

## Introduction

Glioblastoma (GBM) is one of the most common and devastating primary malignant intracranial tumors in human. The current therapy for newly diagnosed GBM is surgical resection followed by radiotherapy plus chemotherapy [1]. However, the prognosis is poor with a median overall survival of only 14.6 months, median progression free survival of 6.9 months and 5 year survival rate of only 9.8% after diagnosis [1, 2]. The treatment failure mainly results from the resistance of malignant glioma cells to current therapeutic modules [3], it is thus in urgent need to identify effective modalities for the management of glioma patients.

Aptamers are designed as 12–30 bases oligonucleotides (ssDNA or RNA), or peptides. They were first identified from basic science studies with viruses in the 1980s and have been found to possess good pharmaceutical properties of drugs [4–5]. Aptamers have increased resistance to serum nucleases and enhanced cellular uptake compared to unstructured molecules. Moreover, quadruplex oligonucleotides are non-immunogenic and heat stable [6]. Therefore, aptamers are promising for the development as drugs for the treatment of various human diseases, including cancers, with numerous aptamers in pre-clinic and clinic trials. AS1411 was developed by Antisoma plc and is the first oligodeoxynucleotide aptamer to reach phase I and II clinical trials for the treatment of cancers, including acute myelogenous leukemia (AML) [7], prostatic cancer [8], and breast cancer [9]. AS1411 can be conjugated with blood-brain barrier (BBB) penetrating peptides which make it a good therapeutic agent for brain tumor [10–11]. Although AS1411 induces cytotoxicity on GBM *in vitro* and *in vivo* [12], the related mechanisms remain unclear. Understanding the effect of AS1411 on glioma may solve drug resistance of GBM and promote further therapeutic strategies.

It has been found that the main pharmacology of AS1411 is to interfere nucleolin (NCL), a protein that has the ability to bind to G-quadruplex-forming DNA sequences [12]. The expression of NCL is correlated with cell proliferative status and its protein level is being widely used as a bio-marker of cell proliferation; moreover, NCL expression has been shown to associate with the development and progression of various cancers [13]. GBM is an aggressive tumor with overexpression of NCL [14]. These facts lead us to speculate that AS1411 may have potential therapeutic effects for GBM via NCL.

In the present study, we investigated the anti-tumor effect of AS1411 on glioma cells both *in vitro* and *in vivo*. The novelty is that we found AS1411 can up-regulate p53 and down-regulate Bcl-2 and Akt1 via NCL hence inhibit growth and proliferation of glioma cells. In mouse GBM xenografts, AS1411 significantly reduced the tumor burden and prolonged the median survival of tumor bearing mice. Our results suggest that AS1411 is a promising agent for the treatment of GBM.

## Materials and Methods

### Ethic statement and patient samples

The study protocol was approved by the Medical Ethics and Human Clinical Trial Committee of First Hospital of Jilin University. All the patients or their relatives have provided written consent to participate in this study. The experiments were performed in accordance with relevant guidelines and regulations. Diagnosis of GBM was histopathologically confirmed by 2 pathologists according to WHO carcinoma. The clinicopathological characteristics of patients were shown in Table 1.

**Table 1:** Clinical characteristics in archival GBM patients.

Factor	Cases
Gender	
Male	22
Female	18
Age (Years)	
< 50	21
≥ 50	19
KPS	
< 75	18
≥ 75	22
Extent of resection	
GTR	25
STR	15
Tumor size (cm)	
< 6	22
≥ 6	18

KPS: Karnofsky Performance Score.

GTR: Gross-total Resection.

STR: Subtotal Resection.

### Cell culture and oligonucleotides

Human glioma U87, U251 cells were obtained from American type culture collection (ATCC). SHG44 cells were obtained from the Cell Bank of the Chinese Academy of Science (Shanghai, China). Normal human astrocytes (NHA) were obtained from four 13-week-old embryos aborted from patients in the First Hospital of Jilin University. NHA cells were differentiated and purified *in vitro* (S1 Fig and S1 File). The glioma cells were grown in Dulbecco’s modified eagle medium (DMEM, Gibco) supplemented with 10% fetal bovine serum (FBS) (Biowest, Nuaillé, France). NHA were cultured with astrocyte media (Invitrogen) containing 10% FBS. Cells were cultured in a humidified incubator maintained at 37°C with 95% air and 5% CO_2_. AS1411, with sequence 5’-d(GGTGGTGGTGGTTGTGGTGGTGGTGG)-3’ and an inactive control oligonucleotide (CRO) (no anti-proliferative activity), 5’d(CCTCCTCCTCCTTCTCCTCCTCCTCC)-3’ as well as 5’-FITC-AS1411, had a phosphodiester backbone and were purchased in the desalted form from Sangon Biotech (Shanghai, China). Aptamers were dissolved in DMEM (without serum) before treatment and stored at -80°C where the solution can be stable for over 6 months.

### Cell viability detection

MTT assay was used the assessment of cell viability. U87, U251, SHG44 and NHA cells were seeded in 96-well plate and exposed to AS1411 at concentration of 0, 2.5, 5, 10μM or 10μM CRO for 24h, 48h, 72h and 96h (NHA for 48h). 20μl MTT (5 mg/ml) (Promega, Shanghai, China) was added to each well and incubated for 4h. Dimethyl Sulphoxide (DMSO) was added to the well after discarding the supernatant. Then, the plate was shaken for 3min. The absorbance was measured at 570nm wavelength using a Microplate Reader (Bio-Rad, Hercules, CA, USA).

### Immunohistochemical staining

Immunohistochemical studies were performed as previously reported [15]. Normal brain tissue was obtained from a frontal lobe trauma patients. Deep gray matter in frontal lobe was used. The sections were incubated with a monoclonal antibody specific for NCL (Santa Cruz, CA) at a 1:100 dilution overnight at 4°C for 24h, and then detected using biotinylated secondary antibodies (Zhongshan Golden Bridge Biotechnology Ltd. Co., China) based on the manufacturer’s protocols. The staining of the slides was carried out by the HRP-streptavidin conjugates. The slides were visualized with diaminobenzidine, and then counterstained with hematoxylin. PBS was used instead of the primary antibodies for the negative controls.

### Flow cytometric assays

Cells were plated at 10^5^ cells per well in six-well plates and exposed to 0, 2.5, 5μM AS1411 or 5μM CRO for 48h. For the detection of cell cycle distribution, cells were fixed in 70% ethanol for 12h at -20°C after collection, then washed twice with PBS, and incubated with 1g/ml propidium iodide (PI) and RNase for 25min. For the detection of cell apoptosis, FITC Annexin V Apoptosis Dectection Kit (BD Pharmingen) was used. Cells were washed with PBS, followed by incubation with PI and FITC Annexin V for 15min according to the manufacturer’s instructions.

### siRNA interference and gene over-expression

The sequences of NCL siRNA duplexes were 5’-GGUCGUCAUACCUCAGAAGtt; The sequences of p53 siRNA duplexes were 5'-CUACUUCCUGAAAACAACGdTdT-3'; 5'-CGUUGUUUUCAGGAAGUAGdTdT-3. The siRNAs were chemically synthesized and annealed by Ambion (Austin, TX) and transfected using X-tremeGENE HP DNA Transfection Reagent (Roche) according to the manufacturer’s directions. The scrambled siRNA used as a negative control was from Ambion. For the gene overexpression, pCEP4 Bcl-2 (Plasmid #16461), pCDH-puro-myr-HA-Akt1 (Plasmid #46969) and GFP-Nucleolin (Plasmid #28176) were obtained from the Addgene (Cambridge, MA). The genes were amplified by PCR and cloned into the corresponding cloning sites in the pcDNA3.1/myc mammalian expression vector. The plasmids were transfected with Lipofectamine 2000 (Invitrogen) following the manufacturer’s instruction. 48 h after transfection, positive transfected cells were selected with 600 μg/ml G418. The Myc tag was detected by western blotting.

### Immunoblotting

Immunoblotting was performed as previously reported [16]. In brief, total proteins were extracted from the cultured cells with radioimmunoprecipitation (RIPA) assay buffer (Cell Signaling Technology, Boston, MA). Samples containing 30–35μg of total protein were loaded onto 8–12% SDS polyacrylamide gel electrophoresis (PAGE), transferred onto a nitrocellulose membrane (Roche), and probed with primary antibodies. Anti-bcl-2, anti-NCL, anti-Akt1, anti-cyclin A1, anti-cyclin B1, and anti-cyclin D1 antibodies were purchased from Santa Cruz Biotechnology (Santa Cruz, CA). Anti-actin antibody (AC-40) was purchased from Sigma-Alderich; Anti-p53 monoclonal antibody (clone Y5) was purchased from abcam (Cambridge, MA). Following incubation with HRP-conjugated goat anti-rabbit, or goat anti-mouse secondary antibodies (ZSGB-BIO), protein bands were visualized by an ECL plus chemiluminescence kit (Beyotime, Haimen, China).

### Immunofluorescence

Immunofluorescence was performed as previously reported [17]. Cells were fixed in 2% formaldehyde and permeabilized in 0.25% Triton X100 in phosphate buffered saline (PBS) for 5 min at room temperature. Cells were then incubated with primary antibodies (anti-NCL), washed in PBS and incubated with the fluorophore-conjugated secondary antibodies. The following secondary antibodies were used: Rhodamine or DyLightTM488 conjugated goat anti-rabbit, fluorescein or DyLightTM594 conjugated goat anti-mouse (ZSGB-BIO). Fluorescence signals were captured by using Olympus Fluoview FV1000 confocal microscope and analyzed by FV10-ASW 1.6 Viewer program (Olympus, Japan).

### Real-time RT-PCR

The final Bcl-2, p53 and NCL primer concentrations were 900nM, and PCR amplification was subjected to 35 cycles of 95°C for 15 sec, 54°C for 2 min. The primer sequences were as following: Bcl-2: (forward) 5-ATGTGTGTGGAGAGCGTCAA-3, (reverse) 5-TAACTATCCTTGCCCGAACG-3 [9]; p53: (forward) 5'-AGGTTGGCTCTGACTGTA-3', (reverse) 5'-CCTCTGTCT-TGAACATGA-3'; NCL: (forward)5’-GCACTTGGAGTGGTGAATCAA A-3’, (reverse) 5’-AAATGCATACCCTTTAGATTTGCC-3’ [9]. Quantitative PCR was done using the SYBR green Jumpstart Taq ReadyMix (TaKaRa) on a Roche LightCycler 480. Reverse transcription involved the Superscript III First Strand Synthesis kit (Invitrogen).

### Invasion and migration assays

The invasion or migration capability of glioma cells was assessed using 24-well transwell containing an 8-mm pore size poly-carbonate membrane with or without matrigel-coated membrane matrix. Cells (2 x 10^5^/ml) were resuspended in 200 μl of serum-free medium. The cells were then plated on the top side of polycarbonate Transwell filter (without matrigel for Transwell assay) or plated on the top side of polycarbonate Transwell filter coated with Matrigel (for Transwell matrix penetration assay) in the upper chamber of the BioCoat Invasion Chambers (BD) and incubated at 37°C for 48 h, followed by removal of cells on the upper chamber with cotton swabs. Migrated and invaded cells on the lower membrane surface were fixed in 4% formaldehyde and stained with 0.1% of crystal violet for 5 min. Randomly selected five fields of cells in each well were counted under a microscope at 200 x magnification.

### Human tumor xenografts in severe combined immunodeficient (SCID) mice

To test the effect of AS-1411 *in vivo*, a xenograft model of human glioma was established. 4-week old male SCID mice were purchased from Vital River Laboratory Animal Technology (Beijing, China). After 1-week acclimatization, mice were subcutaneously injected in the right flank with 5×10^6^ U87 cells re-suspended in 50 ul DMEM media. Treatment was initiated when the subcutaneous tumors reached an average size of 150 to 200mm^3^. Specifically, AS1411 or aptamer control sequence (C-rich sequence; CRO) was subcutaneously injected (adjacent to the tumor area). One injection every 5 days (5μM), for 20 days. Mice received intraperitoneal injection of AS1411 and vehicle as control. Tumor diameter was measured every 2 days with calipers, and the tumor volume was calculated (length×width×height×0.5). Euthanasia was used in the animal experiment to minimized animal suffering and distress. Specific signs indicated “humane endpoints” are ruffled fur, 15–20% weight loss and over 15 mm of tumor diameter. We used 70% CO_2_ for 40 sec to terminate life of animals that reached humane endpoints. We monitored the health of animals every 24 hours and no unexpected death was found. All experiments were carried out in keeping with the procedures and protocols of the Animal Ethics Committee of the First Hospital of Jilin University.

## Results

### High expression of NCL in glioma cells and its high affinity to AS1411

**Fig 1:**
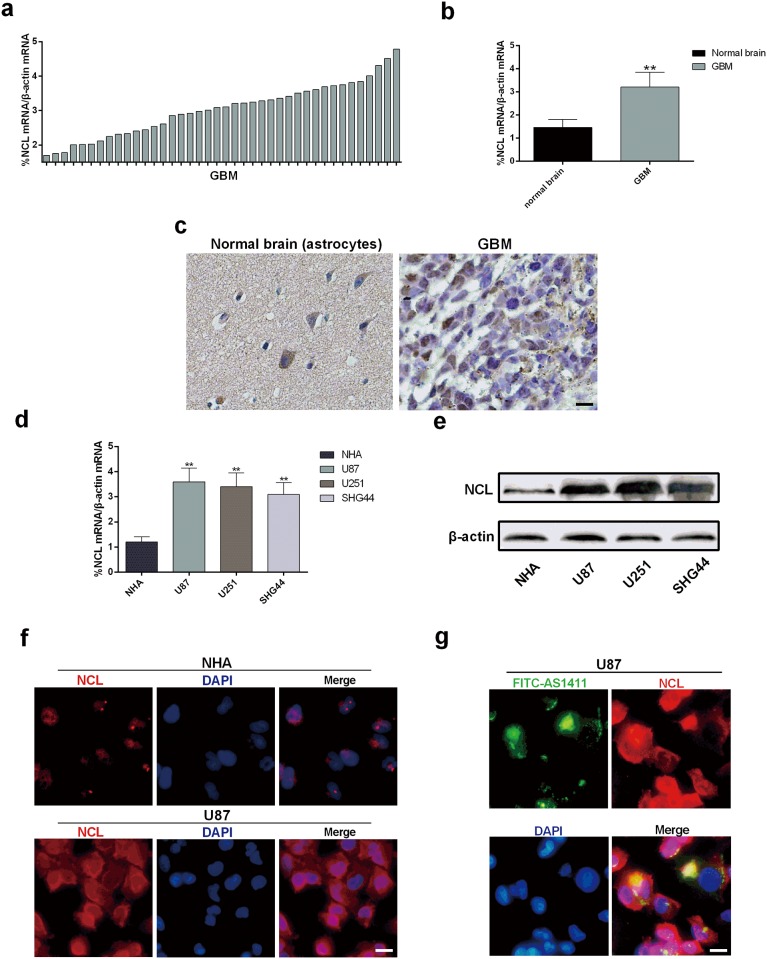
Expression of NCL in glioma cells and NHA and its binding affinity to AS1411. **(a)** Relative mRNA level of NCL in 40 clinical GBM tissues analyzed by Real-time qPCR. **(b)** Real-time qPCR detection of mRNA level of NCL in clinical GBM tissues compared to normal brain tissues **(c)** Immunohistochemical staining of NCL in GBM and normal brain tissues (Astrocytes). Scale bar equals 50 μm. **(d)** Real-time qPCR detection of NCL mRNA in human glioma cells: U87, U251, SHG44 and normal NHA cell. **(e)** Immunoblotting detection of NCL protein in U87, U251 and SHG44 cells compared to NHA, β-actin was used as loading control. **(f)** Immunofluorescence detection of the expression and location of NCL in U87 and NHA cells. **(g)** Immunofluorescence detection of FITC-AS1411 (green) and NCL (red) co-localizations in glioma cells. Error bars indicate ± s.d. *P< 0.05, **P< 0.01, two-tailed student’s t-test. Scale bar equals 20 μm.

It was reported that GBM cells express high level of NCL [18]. In our study, Immunohistochemical staining and Real-time qPCR results demonstrated significantly increased cytoplasmic level of NCL in GBM tissues compared with normal brain samples (Fig 1a–1c). Also, Real-time qPCR showed significantly higher NCL mRNA levels in glioma U87, U251 and SHG44 cells than that in NHA (Fig 1d). Consistent with difference of mRNA levels, immunoblotting analyses demonstrated that NCL was overexpressed in glioma cells when compared to NHA (Fig 1e). To determine the subcellular localization of NCL, immunofluorescence staining of NCL was performed in U87 and NHA cells. The results showed that NCL was highly stained in both nucleus and cytoplasm in U87 cells but only stained in nucleus in NHA (Fig 1f). Similar subcellular localization of NCL was found in breast cancer MCF7 cells and normal epithelial MCF10A cells [9]. Hence, NCL is over-expressed in both nucleolus and cytoplasm in glioma cells.

AS1411 was shown to bind NCL and inhibited the growth of malignant breast cancer cells [9]. To assess the binding of AS1411 to NCL in glioma cells, U87 cells were treated with FITC-AS1411 for 2h. Detection of FITC-AS1411 and NCL was performed by immunofluorescence staining. As shown in Fig 1g, the majority of AS1411 co-localized NCL in both nucleus and cytoplasm in U87 cells. Thus, AS1411 binds NCL [19], suggesting a potential effect of AS1411 in growth inhibition in glioma cells.

### Exposure to AS1411 results in significant growth inhibition in glioma cells compared to NHA

**Fig 2:**
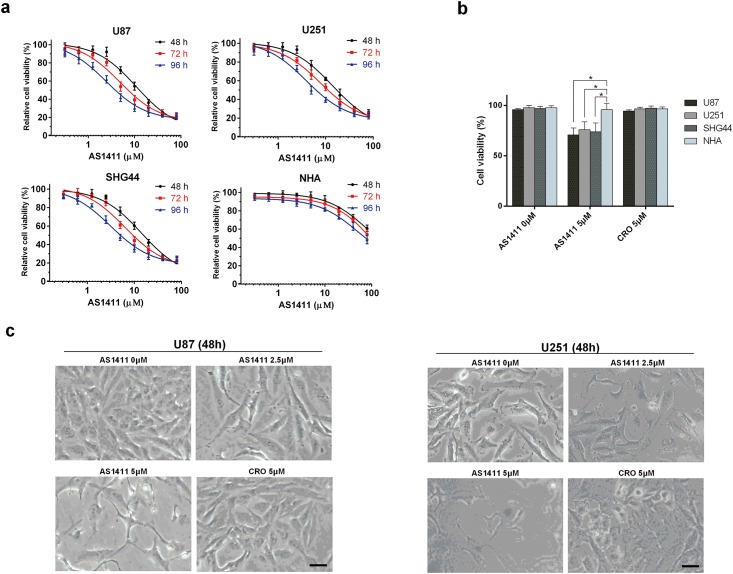
Short term AS1411 exposure induced growth suppression in glioma cells. **(a)** MTT analysis of cell viability after exposure to AS1411 from 0 to 80 μM for 48, 72 and 96h in U87, U251, SHG44 and NHA cells, respectively. **(b)** After 48 h treatment. Cell viability inhibition induced by AS1411 in glioma cells was compared to that in NHA cells. **(c)** 10×Light microscope images indicated the growth inhibition and cell structure destroy in U87 and U251 cells induced by AS1411 of 0 to 5μM and CRO of 5μM for 48 h. Error bars indicate ± s.d. *P<0.05, two-tailed student’s t-test. Scale bar equals 10 μm.

Obvious dose-and time-dependent growth inhibitions induced by AS1411 were observed in U87, U251 and SHG44 cells (Fig 2a). At clinically achievable concentration of AS1411 (5μM) [20], significant cell inhibitory effects were found in glioma cells after exposure to AS1411 (5μM) for 48h (Fig 2a and 2b) but not in NHA. No significant growth inhibition by CRO at 5μM was detected in all the tested cells (Fig 2b). In order to find the effect of AS1411 on cell morphology, the light microscope images of U87 and U251 cells after treatment of AS1411 for 48 h were collected. As shown in Fig 2c, the cell number decreased in the AS1411 group (2.5μM and 5μM) compared to CRO group in both U87 and U251 cells. Besides, AS1411 treated cells demonstrated apopotic cell morphology. These results showed that AS1411 can induce the anti-proliferate effect on glioma cells but not on NHA within a certain range of concentration. Moreover, the inhibitory effect induced by AS1411 on wt-p53 cell lines (U87 and SHG44) is stronger than that on mutant-p53 cell line (U251) after 72 h treatment (S2 Fig).

### AS1411 induces the up-regulation of p53 and down-regulation of Bcl-2 and Akt1 via NCL in glioma cells

**Fig 3:**
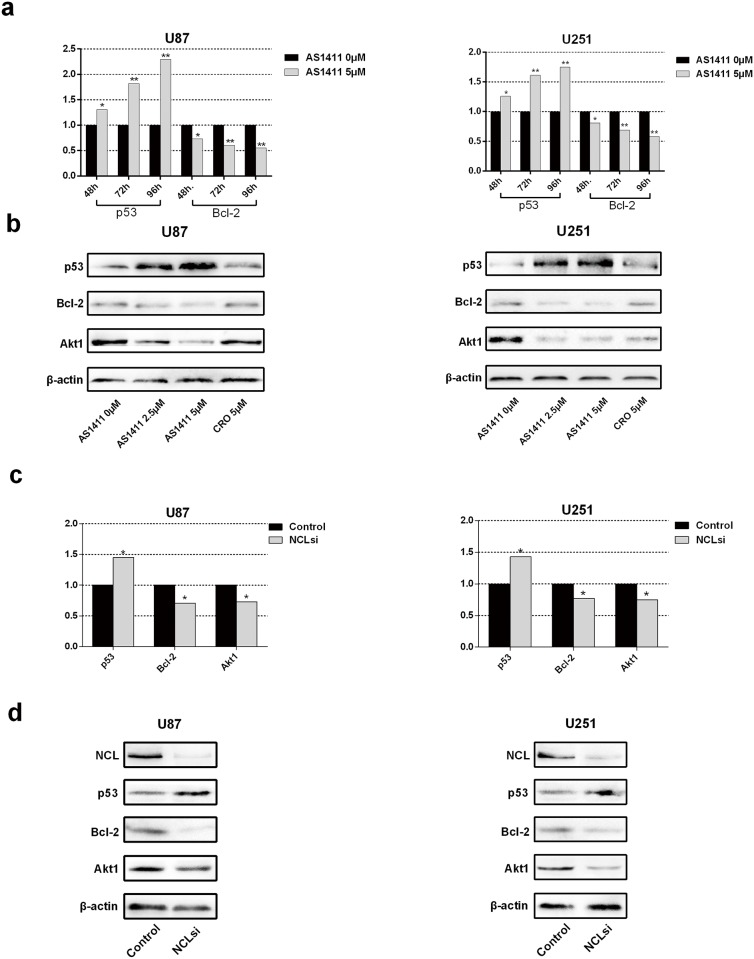
Expression of Bcl-2, p53, Akt1 after AS1411 treatment and NCL silencing in U87 and U251 cells. **(a)** Real-time qPCR detection of Bcl-2, p53 and Akt1 mRNA in U251 and U87 cells after treatment with AS1411 (5 μM) for 48 h, 72 h and 96 h. **(b)** Immunoblotting analysis of Bcl-2, p53 and Akt1 in U251 and U87 cells treated with AS1411 (5 μM) for 48 h, β-actin used as loading control. **(c)** Real-time qPCR detection of Bcl-2, p53 and Akt1 mRNA after siRNA NCL silencing in U87 cells and U251 cells for 48 h. Immunoblotting detection of p53, Bcl-2 and Akt1 after siRNA NCL silencing in U87 and U251 cells for 48 h. **(d)** Error bars indicate ± s.d. *P<0.05, **P< 0.01, two-tailed student’s t-test.

AS1411 has the potential to compete binding the C-terminal region of NCL thereby disabling the function of the NCL to certain mRNAs such as the mRNAs of p53, Bcl-2 and Akt1 [21]. NCL usually binds with the 3'UTR (such as Bcl-2), 5’UTR (p53) and both (Akt 1) terminals of mRNAs. p53 and Bcl-2 play a pivotal role in the regulation of cell death, while Akt1 controls cell cycle, invasion and migration. To explore the mechanism of the growth inhibition by AS1411 in human malignant glioma cells, we examined the mRNA and protein levels of p53, Bcl-2 and Akt1 in U87 and U251 cells following exposure to AS1411. After 48 h, AS1411 treatment significantly increased p53 mRNA and decreased Bcl-2 and Akt1 mRNAs in both U87 and U251 in a time-depended manner (Fig 3a). Similarly, treatment with AS1411 on U87 and U251 cells for 48 h resulted in up-regulation of p53 and down-regulation of Bcl-2 and Akt1 in protein levels in a doze-depended manner (Fig 3b). Further, we found NCL binding 5 UTR of p53 mRNA was antagonized by AS1411, which indicates p53 is transcriptionally regulated by AS1411 via NCL (S3 Fig).

To assess whether the above effects result from disruption of functions of NCL by AS1411, NCL was silenced by siRNA in U87 and U251 cells (S4 Fig). Knockdown of NCL by siRNA led to significant upregulation of p53 mRNA and downregulation of the mRNA levels of Bcl-2 and Akt1 (Fig 3c). In addition, silencing of NCL increased p53 and decreased Bcl-2 and Akt1 proteins in both U87 and U251 (Fig 3d). These results showed that silencing NCL or treatment with AS1411 up-regulated p53 and down-regulated Bcl-2 and Akt1 at both mRNA and protein levels, which indicated a similar effect of the treatment of AS1411 as NCL siRNA. These results suggest that up-regulation of p53 and down-regulation of Bcl-2 and Akt1 is a result of the NCL dysfunction induced by AS1411.

### AS1411 induces G2/M cell cycle arrest via NCL dysfunction in glioma cells

**Fig 4:**
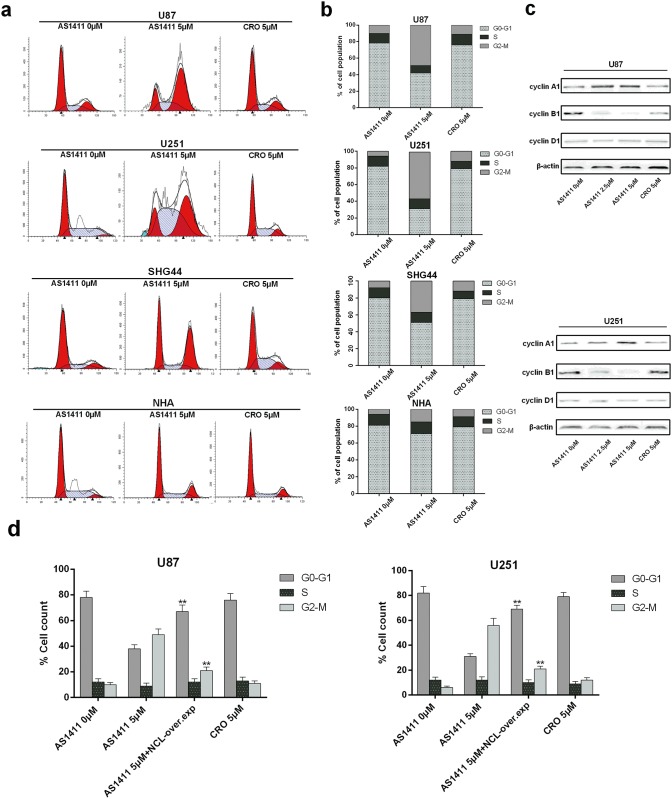
G2/M cell cycle arrest evoked by AS1411. **(a)** Cell cycle arrest induced by AS1411 in U87, U251, SHG44 and NHA cells. 48 h after treatment with AS1411 (5μM), cells were collected and stained with propidium iodide (PI); DNA content was determined by flow cytometry. This assay was performed in triplicate. CRO (5μM) was used as negative control. **(b)** Histograms showing the percentage of glioma cells and NHA in G0-G1, S, and G2-M phases in four independent experiments. **(c)** The G2/M cell cycle related protein cyclin A1, cyclin B1 and cyclin D1 was detected by immunoblotting after treatment with AS1411 (5μM) for 48 h, CRO (5μM) was used as negative control, β-actin was used as loading control. **(d)** Histograms showing the percentage of glioma cells in G0-G1, S, and G2-M phases after the NCL overexpression. Error bars indicate ± s.d. **P<0.01, two-tailed student’s t-test.

A previous study showed that reduction of NCL expression through siRNA-mediated knockdown in the U87 cell caused a dramatic decrease in cell proliferation and induced cell cycle arrest *in vitro* [22]. The similar patterns of the up-regulation of p53 and down-regulation of Bcl-2 and Akt1 by NCL siRNA and AS1411 suggest AS1411 may have the ability to cause cell cycle arrest in glioma cells. To this end, U87, U251, SHG44 and NHA cells were exposed to 5μM AS1411 or 5μM CRO for 48h and cell cycle distribution was analyzed with PI staining and flow cytometry. AS1411 induced an increase of cells in G2-M phase with concomitant decrease of cells in the G0 and G1 phases in U87, U251 and SHG44 but not NHA cells (Fig 4a and 4b). Furthermore, AS1411 increased the protein levels of cyclin A1 and decreased the protein levels of cyclin B1 in both U87 and U251 cells. No obvious alteration of cyclin D1 by AS1411 was observed in both cell lines (Fig 4c). Interestingly, overexpression of NCL significantly prevented induction of the G2/M cell cycle arrest by AS1411 in both U87 and U251 cells (Fig 4d). These results indicated that AS1411 is able to perturb the cell cycle progression of glioma cells by inducing NCL dysfunction.

### AS1411 induces apoptosis in a p53 and Bcl-2 dependent manner in glioma cells

**Fig 5:**
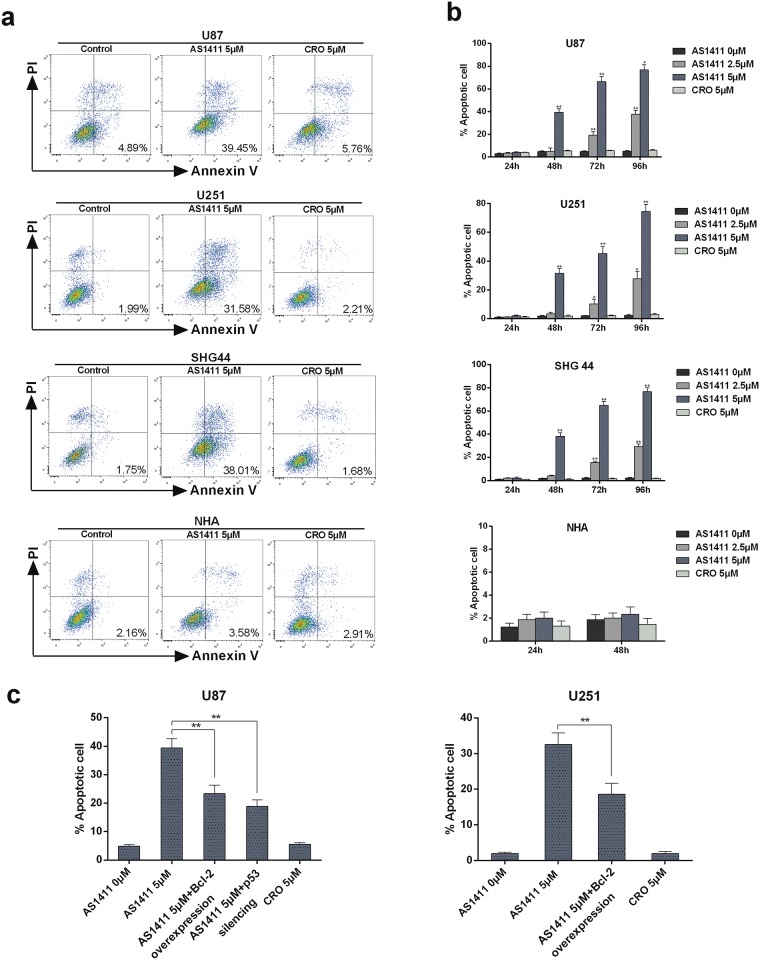
Apoptosis induced by AS1411 in glioma cells. **(a)** Apoptotic cell death induced by AS1411 in U87, U251, SHG44 and NHA cells. 48h after treatment, cells were collected and stained with PI and Annexin V–FITC, Annexin V-positive/PI-negative cells were measured by flow cytometry. This experiment was repeated three times. **(b)** Histograms showing the percentage of cells in apoptosis. U87, U251, SHG44 and NHA cells in four independent experiments. Annexin V-positive cells were considered as apoptotic cells. **(c)** Histograms showing the percentage of glioma cells apoptosis after the Bcl-2 overexpression and p53 siRNA silencing. Error bars indicate ± s.d. **P<0.01, two-tailed student’s t-test.

We explored whether NCL interference by AS1411 results in cell apoptosis in glioma cells. Firstly, we performed the staining of cells with Annexin V and PI, followed by analysis with flow cytometry. The proportion of apoptotic cells with Annexin V staining was increased in a dose-dependent manner in U87, U251 and SHG44 cells compared to NHA after treatment of AS1411 for 48h (Fig 5a). In addition, AS1411 increased Annexin V staining in a time-dependent way in U87, U251 and SHG44 cells but not in NHA cells (Fig 5b). However, overexpression of Bcl-2 significantly reduced the induction of apoptosis by AS1411 in both U87 and U251 cells. Silencing of p53 by siRNA also inhibited cell apoptosis in U87 cell (Fig 5c). Further research is needed to access whether silencing of p53 can result the same effect in U251 since U251 is one of the p53-mutant cell lines [23]. It has been reported that AS1411 reduced NCL binding to Bcl-2 mRNA resulting in BCL2 mRNA destabilization and downregulation of Bcl-2 protein levels [9, 24–25]. Besides, Bcl-2 is also repressed by p53 [26]. Our results suggest AS1411 may induce cell apoptosis in glioma cells by influencing p53 and Bcl-2 mRNA via NCL.

### AS1411 inhibits cell migration and invasion of glioma cells *in vitro* by down regulation of Akt1

**Fig 6:**
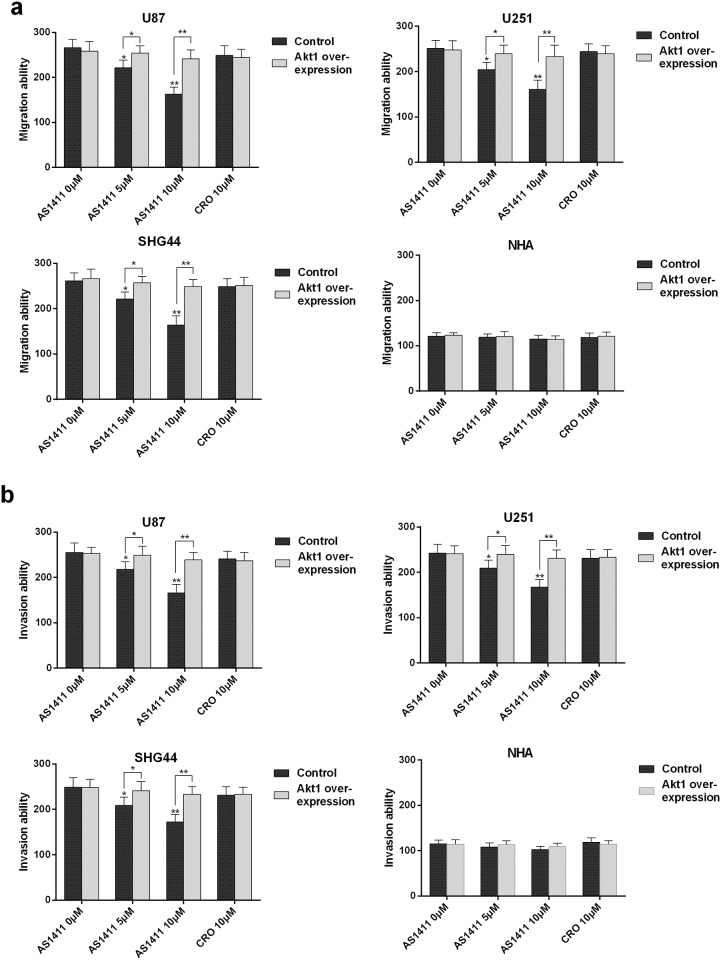
Migration and invasion inhibition induced by AS1411. **(a)** AS1411 inhibited the migration of glioma cells in vitro. The migration capabilities of U87, U251, SHG44 and NHA cells were assessed after pretreatment with AS1411 of 0 to 10 μM and CRO of 10μM. Akt1 overexpression antagonized AS1411 induced migration inhibition. **(b)** AS1411 inhibited the invasion of glioma cells in vitro. The invasive capabilities of U87, U251, SHG44 and NHA were assessed after pretreatment with AS1411 of 0 to 10μM and CRO of 10μM. Akt1 overexpression antagonized AS1411 induced invasion inhibition. Error bars indicate s.d. *P<0.05, **P<0.01, two-tailed Student’s *t*-test.

Previous studies showed that Akt1 mRNA has the ability to bind to NCL [27]. The activation of AKT promotes glioma invasiveness, angiogenesis and migration. The suppression of the expression of Akt1 by AS1411 (Fig 3a and 3b) suggests that AS1411 may reduce the glioma invasiveness and metastasis. Indeed, AS1411 significantly reduced the migration of U87, U251 and SHG44 cells in a dose dependent manner but not NHA cells (*P*<0.05; Fig 6a). Further, AS1411 significantly reduced the invasion ability of U87, U251 and SHG44 cells in a dose dependent way but not NHA cells (*P*<0.05; Fig 6b). In sharp contrast, ectopic overexpression of Akt1 significantly abolished the suppression of the migration (*P*<0.05; Fig 6a) and invasion (*P*<0.05; Fig 6b) of U87, U251 and SHG44 cells. These results indicate that AS1411 inhibits glioma cell migration and invasion by reducing Akt1 in glioma cells.

### AS1411 has anti-tumor activity in xenograft models

**Fig 7:**
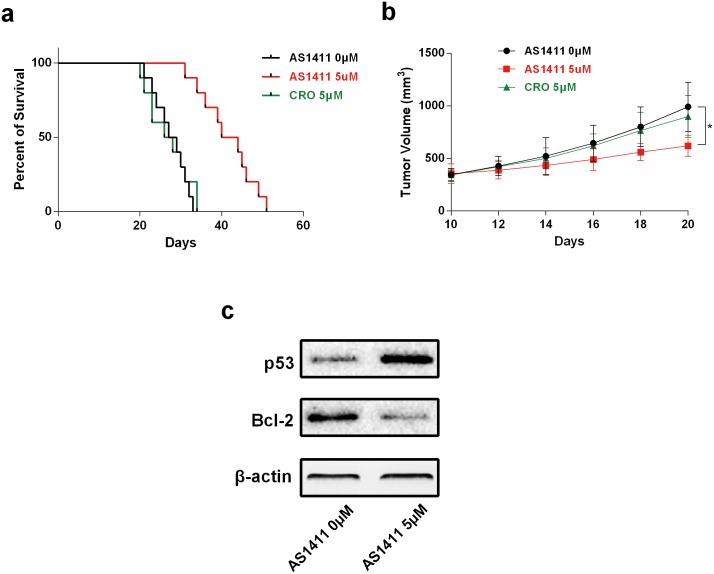
Tumor growth inhibition and life expansion induced by AS1411 *in vivo*. Subcutaneous tumors generated from U87 cells were allowed to reach a volume of 200 mm^3^ and were treated with AS1411 and CRO. **(a)** Survival of brain tumor–bearing mice was recorded and represented in a Kaplan–Meier plot. **(b)** Tumor volume during the course of treatment. **(c)** AS1411 treatment (20 days) induced down-regulation of Bcl-2 and up-regulation of p53 protein in tumor tissue. *P<0.05, **P<0.01, two-tailed Student’s t-test.

To examine anti-tumor effects of AS1411 *in vivo*, a human glioma-SCID mouse model using the U87 cell was established. As shown in Fig 7a, AS1411 treatment (25mg/kg) remarkably prolonged the survival of tumor-bearing mice (*P*<0.05). In addition, the mice that received AS1411 treatment had a lower tumor burden as compared with the control group (*P*<0.05; Fig 7b). These results demonstrated AS1411 inhibited the growth of U87 xenograft and prolonged the survival time of glioma tumor-bearing mice. Further, we found a down-regulation of Bcl-2 and up-regulation of p53 protein in tumor tissue after treatment with AS1411 (Fig 7c).

## Discussion

AS1411 was reported to have the ability to bind NCL [28, 29]. In this study, we demonstrated that NCL was over-expressed in glioma cells and tissues. AS1411 co-localized NCL and inhibited the proliferation, migration and invasion of glioma cells, accompanied with induction of apoptosis and cell cycle arrest at G2/M phase. Moreover, either silencing NCL or AS1411 up-regulated p53 while down-regulated Bcl-2 and Akt1. Importantly, AS1411 inhibited the growth of U87 xenograft and prolonged the survival time of glioma tumor-bearing mice. Our results indicated that AS1411 inhibited the growth of glioma cells via disrupting the functions of NCL, suggesting a promising further development of AS1411 for the treatment of glioma patients in clinic.

NCL presents in the nucleoli, nucleoplasm, cytoplasm, and plasma membrane of cells [30–31]. It plays an important role in ribosome biogenesis, which include the control of rDNA transcription, pre-ribosome packaging, and organization of nucleolar chromatin. NCL is also a shuttle protein that transports viral and cellular proteins between the cytoplasm and nucleus/nucleolus. NCL has also been implicated, directly or indirectly, in the regulation of many other biology processes including apoptosis, nuclear matrix structure, DNA replication, mRNA stability, transcriptional regulation, signal transduction, telomere maintenance, cytokinesis, as a nucleic acid helicase, and as a G-quadruplex binding protein. In addition, there are numerous reports describing the presence of NCL on the cell surface and its function as a receptor for a variety of ligands [19, 32–33]. Because of the multifunctional nature of NCL, it has been proposed that many secondary targets are affected following treatment with AS1411 [34]. NCL is higher expressed in tumor cells than in normal cells [35–36], besides, NCL expresses in cell membrane, cytoplasm and nucleus in tumor cells while only within nucleus in normal cells [29, 37], AS1411 binds NCL on external surface of the cell and then is internalized [9]. Expressing only nucleus NCL, NHA is weakly respond to AS1411 mainly due to the limited internalization and interaction with NCL.

NCL has been reported to have the ability to interfere with DNA and mRNA of several proteins [38–39]. The C-terminal region of NCL contains anarginine/glycine-rich domain (RGG), through which NCL can interact with target mRNAs as well as other proteins like ribosomal proteins [40–41]. The sequence of AS1411 is similar to many NCL target mRNAs, suggesting AS1411 has the potential to compete binding the C-terminal region of NCL [21]. Many of NCL target mRNAs bear AU-rich elements (AREs), typically present in their 3'-untranslated region (UTR), and/or G-rich sequences distributed in the 5' UTR, coding region (CR), and 3' UTR [42]. Our study showed NCL siRNA silencing resulted in the down-regulation of Bcl-2 and up-regulation of p53, which may be explained by the interaction between NCL and 3’ UTR of Bcl-2 mRNA [24] and 5’ UTR of p53 mRNA [43]. The binding between NCL and Bcl-2 mRNA promotes the expression of proto-oncogene Bcl-2, which blocks apoptosis in cancer cells [24], while the binding between NCL and p53 mRNA reduces the translation of the pro-apoptotic tumor suppressor p53, further enhancing an anti-apoptotic cell phenotype [43–44]. We found that treatment of AS1411 and NCL silencing by siRNA had the same effect on the expression of Bcl-2 and p53 in glioma cells. The up-regulation of p53 and down-regulation of Bcl-2 is responsible for the cell apoptosis. Our results showed that the induction of apoptosis by AS1411 was abolished by the over-expression of Bcl-2 and silencing of p53. Our findings suggest that the interaction of AS1411 to the binding site of NCL lead to the up-regulation of p53 and down-regulation of Bcl-2.

Literature previously reported the enhanced translation of a subset of target mRNAs by interacting with G-rich elements present in both CR and UTRs [45–47]. These mRNAs include those that encode the pro-survival proteins Akt1. By binding to Akt1 mRNA, NCL enhances the translation of Akt1 to promote the survival of the malignant cells. In this study, we showed that both treatment of AS1411 and NCL silencing by siRNA decreased Akt1 in glioma cells, which is consistent with previous studies [48–49], and suggest the down-regulation of the Akt1 is one of the mechanisms that responsible for AS1411-induced migration and invasion inhibition of glioma cells.

Previous studies has shown that the siRNA knockdown of NCL leads to cell cycle arrest in HeLa cells [50], and in U87 cells [22]. Also, AS1411 can induce an S-phrase cell cycle arrest in gastric cancer cells [51] and PC3 cancer cells [52]. While in our study, obvious G2/M arrest was induced by AS1411 in U87 and U251 cells. The C-terminal of NCL was reported to have the ability of regulate the cell cycle progression [32]. Considering the same effects induced by AS1411 and NCL siRNA, we evaluated the effect of AS1411 on the cell cycle progression and revealed AS1411 induced G2/M cell cycle arrest, accompanied with the up-regulation of cyclin A1 and down-regulation of cyclin B1, which are responsible for the G2/M cell cycle arrest [32].

Preclinical mouse models have shown a great promise of AS1411 for the treatment of several solid tumors [53]. Our results showed a reduction of tumor volume and extension of survival period in glioma xenograft models, suggesting a promising of AS1411 in the future clinical application in the therapy of glioma. In phase I trial, AS1411 has been tested without serious systemic toxicity to patients. Moreover, AS1411 can be heated to 80°C or stored in various solvents/harsh environments, and will return to their original conformation. Furthermore, aptamers may penetrate tumors better and be more rapidly cleared from the blood. Notably, AS1411 also appears to bind to NCL in cell surface specifically, and is subsequently internalized into the tumor cell. We therefore propose AS1411 as possible adjuvant agent to the standard therapeutic protocols presently utilized for glioma.

In conclusion, AS1411 induced cell apoptosis and cycle arrest, and inhibited cell viability by up-regulation of p53 and down-regulation of Bcl-2 and Akt1 in human glioma cells compared to NHA cell. Most importantly, AS1411 inhibited the growth of mouse glioma xenograft and prolonged the survival time of glioma tumor-bearing mice. Furthermore, targeting NCL means AS1411 may induce a stronger curative effect on high grade glioma which expresses higher level of NCL [19, 54]. In all, our results suggest a promising further development of AS1411 for the treatment of glioma patients in clinic.

## Supporting Information

S1 Fig Differentiation of astrocyte from stem cells. **(a)** Immunofluorescence detection of glial fibrillary acidic protein (GFAP) positive cells. **(b)** Percentage of GFAP-positive cells in total cells after treatment with trypsin. (Scale bar = 10μm).
(TIF)

S2 Fig AS1411 induced growth suppression in wt-p53 cell lines vs mutant-p53 cell line. MTT analysis of cell viability after exposure to AS1411 5μM for 48 and 72h in U87, U251, SHG44 cells, respectively. Though significant growth inhibitions were found in all of the 3 cell lines (Fig 2), AS1411 induced a stronger growth suppression in wt-p53 cell lines (U87 and SHG44) compared to mutant-p53 cell line (U251) in 72 h after treatment. *P<0.05, two-tailed student’s t-test.
(TIF)

S3 Fig RNA pull down assay. Whole cell extract (WCE) prepared from U87 cell after treatment with AS1411 5μM for 48 h, and the binding of nucleolin protein to biotinylated p53 5’ UTR was tested. Then the bound fractions are analyzed by immunoblotting.
(TIF)

S4 Fig siRNA knockdown of Nucleolin. **(a)** Immunoblotting detection of NCL in nuclear extract (NE), cytosolic extract (CE) and whole cell extracts (WCE) after NCLsi. **(b)** Then the percentage of NCL protein in nuclear, cytosolic and whole cell extracts after NCLsi compared with control group (100%) was calculated.
(TIF)

S5 Fig Tumor volume analysis after AS1411 treatment for 30 days. Tumor volume decreased significantly after treatment with AS1411 5μM for 30 days. **P<0.01, two-tailed student’s t-test.
(TIF)

S1 File Supplementary Methods. (DOCX)
