# Supplementary material for: AS1411-Induced Growth Inhibition of Glioma Cells by Up-Regulation of p53 and Down-Regulation of Bcl-2 and Akt1 via Nucleolin
Source: PLoS One. 2016 Dec 1;11(12):e0167094. doi: 10.1371/journal.pone.0167094 (PMC5132312; doi:10.1371/journal.pone.0167094)
Supplement: S1 File — (DOCX) [file pone.0167094.s006.docx]

**Supplementary Methods**

**Normal human astrocytes (NHA) preparation**

NHA were obtained from four 13-week-old embryos aborted from patients in the First Hospital of Jilin University. 12 days after plating, a rotary shaker was used to shake the primary cultures so that to remove the microglia and fibroblasts. After 4 week’s culturing, cultures were shaken again and trypsin was used for astrocyte isolation. Then we passaged the cells for 3 generation, Immunofluorescence was used to detect the glial fibrillary acidic protein (GFAP) which can be detected in astrocytes but not in microglial cells [55-58]. Cells were qualified for further experiments when more than 90% is GFAP-positive (S1 Fig).

**Tissue protein extraction**

Tumors removed from the animals at the end of experiments were transferred and stored in liquid nitrogen for future use. Active protein from the tumor tissue was extracted using Tissue Active Protein Extraction Kit (Sangon Biotech, Inc. China). Immunoblotting detections of p53 and Bcl-2 are described in Method section.

**RNA pull down assay**

P53 5’ UTR RNA was generated and synthesized in vitro using T7 polymerase (New England Biolab, Inc. USA) and labeled with biotinylated uridines [59]. U87 cells were exposed to AS1411 of 0 and 5 mM for 48 h and then were treated with RIPA Lysis Buffer (Sigma Aldrich, USA). Total cell extract was incubating with streptavidin beads for 0.5 hour at 4 C with rotation. Then add the biotinylated RNA and incubate for another 1 hour. The beads was washed for 5 times and then boiled in protein loading buffer for immunoblotting. hnRNP-U was detected by anti- hnRNP-U antibody (Abcam, Inc. USA) as positive control. Immunoblotting detection of NCL protein is described in Method section.

**References**

55. Janabi N, Mirshahi A, Wolfrom C, Mirshahi M, Tardieu M. Effect of interferon gamma and TNF alpha on the differentiation/activation of human glial cells: implication for the TNF alpha receptor 1. Res Virol 1996; 147: 147-153.

56. Héry C, Sébire G, Peudenier S, Tardieu M. Adhesion to human neurons and astrocytes of monocytes: the role of interaction of CR3 and ICAM-1 and modulation by cytokines. J Neuroimmunol 1995; 57: 101-109.

57. Deiva K, Khiati A, Hery C, Salim H, Leclerc P, Horellou P, et al. CCR5-, DC-SIGN-dependent endocytosis and delayed reverse transcription after human immunodeficiency virus type 1 infection in human astrocytes. AIDS Res Hum Retroviruses 2006; 22: 1152-1161.

58. Kleiderman S, Gutbier S, Ugur Tufekci K, Ortega F, Sá JV, Teixeira AP, et al. Conversion of Nonproliferating Astrocytes into Neurogenic Neural Stem Cells: Control by FGF2 and Interferon-γ. Stem Cells. 2016 Sep 7. doi: 10.1002/stem.2483. [Epub ahead of print]

59. Takagi M, Absalon MJ, McLure KG, Kastan MB. Regulation of p53 translation and induction after DNA damage by ribosomal protein L26 and nucleolin. Cell 2005; 123: 49-63.
